# Prognostic Value of NLRP3 Inflammasome and TLR4 Expression in Breast Cancer Patients

**DOI:** 10.3389/fonc.2021.705331

**Published:** 2021-09-02

**Authors:** Concetta Saponaro, Emanuela Scarpi, Margherita Sonnessa, Antonella Cioffi, Francesca Buccino, Francesco Giotta, Maria Irene Pastena, Francesco Alfredo Zito, Anita Mangia

**Affiliations:** ^1^Functional Biomorphology Laboratory, IRCCS Istituto Tumori “Giovanni Paolo II”, Bari, Italy; ^2^Unit of Biostatistics and Clinical Trials, IRCCS Istituto Scientifico Romagnolo per lo Studio dei Tumori (IRST) “Dino Amadori”, Meldola (FC), Italy; ^3^Pathology Department, IRCCS Istituto Tumori “Giovanni Paolo II”, Bari, Italy; ^4^Medical Oncology Unit, IRCCS-Istituto Tumori “Giovanni Paolo II”, Bari, Italy

**Keywords:** NLRP3 inflammasome, breast cancer, prognostic biomarker, PYCARD, TLR4

## Abstract

Inflammasome complexes play a pivotal role in different cancer types. NOD-like receptor protein 3 (NLRP3) inflammasome is one of the most well-studied inflammasomes. Activation of the NLRP3 inflammasome induces abnormal secretion of soluble cytokines, generating advantageous inflammatory surroundings that support tumor growth. The expression levels of the NLRP3, PYCARD and TLR4 were determined by immunohistochemistry in a cohort of primary invasive breast carcinomas (BCs). We observed different NLRP3 and PYCARD expressions in non-tumor *vs* tumor areas (p<0.0001). All the proteins were associated to more aggressive clinicopathological characteristics (tumor size, grade, tumor proliferative activity etc.). Univariate analyses were carried out and related Kaplan-Meier curves plotted for NLRP3, PYCARD and TLR4 expression. Patients with higher NLRP3 and TLR4 expression had worse 5-year disease-free survival (DFS) compared to patients with lower NLRP3 and TLR4 expression (p =0.021 and p = 0.009, respectively). In multivariate analysis, TLR4 was confirmed as independent prognostic factors for DFS (HR = 2.03, 95% CI 1.16–3.57, p = 0.014), and high NLRP3 expression showed a slight association with DFS (HR = 1.75, 95% CI 0.98–3.15, p = 0.06). In conclusion, we showed TLR4 expression as independent prognostic factors and we highlighted for the first time that high expression of NLRP3 is linked to a poor prognosis in BC patients. These results suggest that NLRP3 and TLR4 could be two new good prognostic factor for BC patients.

## Introduction

The tumor microenvironment (TME) is the result of a multistep process characterized by reprogramming across cellular components. Different critical oncogenic processes contribute to changing the TME, such as angiogenesis, invasion/metastasis, drug resistance and chronic inflammation (1). Uncontrolled chronic inflammation has been shown to play a pivotal role in the onset and development of cancer *via* the up-regulation of growth factors, free radicals, prostaglandins and pro-inflammatory cytokines [interleukin (IL)-1β and IL-18] (2, 3). Tumor cells can also produce inflammatory mediators as well as fibroblasts, immune and endothelial cells (4). The inflammatory microenvironment can contribute to enhancing mutational state and mutated cell proliferation. However, the triggers and molecular signaling implicated in the inflammatory state are still poorly characterized.

The production of inflammatory interleukins is mediated by inflammasome activation. Inflammasomes are cytosolic multiprotein oligomers involved in the inflammatory state. They typically comprise a sensor of a NOD-like receptor protein (NLRP), the adaptor molecule apoptosis-associated speck-like protein containing a CARD (ASC) and a pro-caspase (5). Inflammasomes are activated by different stimuli mediated by diverse Pathogen Recognition Receptors (PRRs), including Toll-like receptors (TLRs), NOD-like receptor (NLRs), and Absent in melanoma-like receptors (ALR) (6). TLRs and NLRPs are intercellular receptors that are able to identify stimuli called pathogen-associated molecular patterns (PAMPs) and danger-associated molecular patterns (DAMPs) (7, 8).

Inflammasome components are involved in different physiological and pathological conditions, and their contribution in different cancers has been highlighted in the past few years (9–12). The role of inflammasomes in cancer is dual. On the one hand, inflammasome activation accelerates tumor progression by enhancing cancer stem cells, myeloid-derived suppressor cells (MDSCs), metastasis, epithelial mesenchymal transition (EMT) and angiogenesis and inhibiting apoptosis (13, 14). This behavior makes inflammasomes the perfect structure bridging chronic inflammation, carcinogenesis and tumor progression. On the other hand, inflammasomes can constrain tumor cell survival by supporting tumor suppressors and immune response and promoting cell death by pyroptosis (9, 15, 16). However, inflammasomes are not the only complex with a dual role and the TME, tissue type and cell type are also involved in determining oncogene and onco-suppressor behavior.

The NLRP3 inflammasome is the most well-studied inflammasome involved in cancer development. Its role in breast cancer (BC) is becoming clearer, but several aspects have yet to be analyzed. Recent papers have associated NLRP3 activation and IL-1β secretion to tumor growth, invasiveness, relapse and progression (13, 17–19). An association has also been reported between levels of TLR expression and high recurrence rates in BC patients (20), and high messenger RNA (mRNA) levels of TLR3, TLR4, and TLR9 have been observed in BC (21).

The aim of our study has been to clarify the role of some proteins of the NLRP3 inflammasome platform in a cohort of women with primary invasive BC and identify new potential prognostic biomarkers to determine a sub-group of patients who may benefit from specific treatments.

## Material and Methods

### Patients and Clinicopathological Characteristics

A retrospective, non-consecutive series of 374 patients with confirmed primary invasive BC from the Istituto Tumori “Giovanni Paolo II” of Bari, Italy was studied. The patients were selected based on the availability of biological material and their clinical follow-up. Patients were eligible if they had a histological diagnosis of invasive breast carcinoma of any size and no evidence of metastatic disease at diagnosis. The study was approved by the Ethics Committee of the Istituto Tumori “Giovanni Paolo II” with document no. 234/CE of 13 November 2017. **Table 1** summarizes the clinicopathological characteristics of the entire cohort. One hundred and one (31.7%) were triple negative breast cancers (TNBCs). Median age was 53 years (IQR=interquartile range 46-63) and median follow-up was 67 months (range 1-199). Sixty patients (17%) had a relapse. The tumor, node, metastasis (TNM) classification, tumor size, histological grade, estrogen receptor (ER) status, progesterone receptor (PR) status, proliferative activity (Ki67 expression and human epidermal growth factor receptor 2 (HER2) status were provided by the Pathology Department of our Institute. The immunohistochemical assessment of ER status, PR status and Ki67 expression has been previously reported (22). Cases scoring 0 and 1+ were classified as negative. HER2 was considered to be positive if immunostaining was 3+ or if a score of 2+ showed gene amplification by fluorescence *in situ* hybridization (FISH), according to the 2007 ASCO/CAP guideline for BC (23).

**Table 1:** Tumor characteristics of 352 invasive breast cancer patients.

	N. (%)
**Age (years):** median value (range, IQR)	53 (29-80, 46-63)
≤53	182 (51.7)
>53	170 (48.3)
**Histotype**	
IDC	315 (90.0)
ILC	21 (6.0)
Other	14 (4.0)
unknown	2
**Tumor size (cm)**	
≤2.0	178 (51.3)
>2.0	169 (48.7)
Unknown	5
**Node**	
Negative	208 (59.6)
Positive	141 (40.4)
unknown	3
**Grade**	
1	13 (3.7)
2	137 (39.3)
3	199 (57.0)
unknown	3
**ER (%)**	
≤10	147 (41.9)
>10	204 (58.1)
unknown	1
**PgR (%)**	
≤10	185 (52.7)
>10	166 (47.3)
unknown	1
**Ki67 (%)**	
≤20	141 (40.5)
>20	207 (59.5)
unknown	4
**HER2**	
Negative	289 (83.3)
Positive	58 (16.7)
unknown	5
**TNBC**	
** No**	237 (68.3)
** Yes**	110 (31.7)
**NLRP3**	
Negative (<80%)	227 (68.2)
Positive (≥80%)	106 (31.8)
unknown	19
**PYCARD**	
Negative (<20%)	205 (61.4)
Positive (≥20%)	129 (38.6)
unknown	18
**TLR4**	
Negative (<20%)	208 (64.4)
Positive (≥20%)	115 (35.6)
Unknown	29

IQR, interquartile range; IDC, Invasive ductal carcinoma; ILC, Invasive lobular carcinoma; ER, Estrogen receptor; PR, Progesterone receptor; HER2/neu, Human epidermal growth factor receptor 2; NLRP3, NOD-like receptor protein 3; PYCARD, Apoptosis-Associated Speck-Like Protein Containing a Pyrin and CARD domain; TLR4, Toll-like receptor 4; TNBC, triple negative breast cancer.

### Tissue Microarrays and Immunohistochemistry

Tissue microarrays (TMAs) were prepared, and immunohistochemistry (IHC) was performed as previously reported. Briefly, TMAs were assembled from formalin-fixed and paraffin-embedded (FFPE) tumor tissues using the Galileo Tissue MicroArrayer CK 4500 (Transgenomic, Hillington Park, Glasgow, UK). Each sample was arrayed in triplicate to minimize tissue loss and to overcome tumor heterogeneity. Consecutive sections of 4-µm thickness were cut from formalin-fixed and paraffin-embedded histological material and stained with an indirect immunoperoxidase method using the BenchMark XT automated staining instrument (Ventana Medical Systems, Tucson, AZ, USA), as previously reported (24). Deparaffinization was performed with EZ PREP solution, followed by antigen retrieval with Cell Conditioning solution 1 at 95° for NLRP3 (32 min) and TLR4 (60 min), and Cell Conditioning solution 2 at 95°C for PYCARD (32 min). The slides were then incubated at 37° for 1h with the specific primary antibody as reported in **Table S1**. The OptiView DAB IHC Detection Kit and OptiView Amplification Kit (Ventana Medical Systems, Tucson, AZ, USA) were used to detect NLRP3 and PYCARD protein expression. The UltraView Universal DAB detection kit (Ventana Medical Systems, Tucson, AZ, USA) was used to detect TLR4 protein expression. Finally, tissues were counterstained with hematoxylin and a bluing reagent for 8 min and 4 min respectively and were then dehydrated and mounted. Positive and negative controls were included in each staining run as indicated in the datasheet of each antibody. All the antibodies used in this study have been validated in the pre-analytic phase to guarantee a satisfactory level of reproducibility and accuracy. All the solutions were from Ventana Medical Systems unless otherwise specified.

### Immunohistochemical Assessment

Cytoplasmic expression of NLRP3, PYCARD and TLR4 was considered. For all biomarkers, the best cutoff values of protein expressions were determined using the receiver operating characteristic (ROC) curve analysis to predict DFS at 5 years. For NLRP3 the best cutoff was 80%, for PYCARD and TLR4 the best cutoff was 20%. All stained specimens were independently assessed by two observers blind to the clinicopathological data. Three distinct visual fields were selected to evaluate the slides using x400 magnification in a bright field microscope (Leica, DMLB). Discordant scores were reviewed and resolved by discussion. Non tumor (NT) counterparts were also evaluated.

### Follow-up and Statistical Analysis

Disease-Free Survival (DFS) was defined as the time from the date of surgery to the date of first relapse or progression of disease or to the date of a second invasive breast cancer/secondary primary cancer and/or death without evidence of breast cancer or to the date of the last follow-up. Overall Survival (OS) was defined as the time between the date of surgery and the date of death from any cause or the date of the last follow-up.

Time-to-event variables were estimated using the Kaplan-Meier method and comparisons between curves were done using the Log-rank test.

In order to identify the prognostic factors for DFS and OS, univariable and multivariable Cox regression models were used to estimate hazard ratios (HR) and their 95% Confidence Intervals (95% CI).

For the expression analysis of NT versus tumor (T) tissues two-tailed non-parametric Kruskal–Wallis and Mann–Whitney U-tests were performed. The association of baseline factors and protein expressions was evaluated with the Chi-square test, while the correlation between continuous variables was evaluated with the Spearman correlation test.

All tests were two sided and p<0.05 were considered to be statistically significant. Statistical analyses were performed using the Prism version 5.00 software package (Graph-Pad Software, San Diego, CA, USA) and SAS statistical software version 9.4 (SAS Institute Inc., Cary, NC, USA).

## Results

### Protein Expression Profiling of NLRP3, PYCARD and TLR4

High NLRP3, PYCARD and TLR4 expression was found in 31.8% (106/333), 38.6% (129/334) and 35.6% (115/323) of the tumor samples, respectively (**Table 1**).

NLRP3, PYCARD and TLR4 expression was evaluated according to their specific cut-off as described in the Material and Methods section. All the three proteins were also evaluated in the NT counterparts, if available. High NLRP3, PYCARD and TLR4 expression was found in 1.5% (1/67), 0% (0/82) and 3.8% (10/26) of the NT samples, respectively.

**Figure 1:**
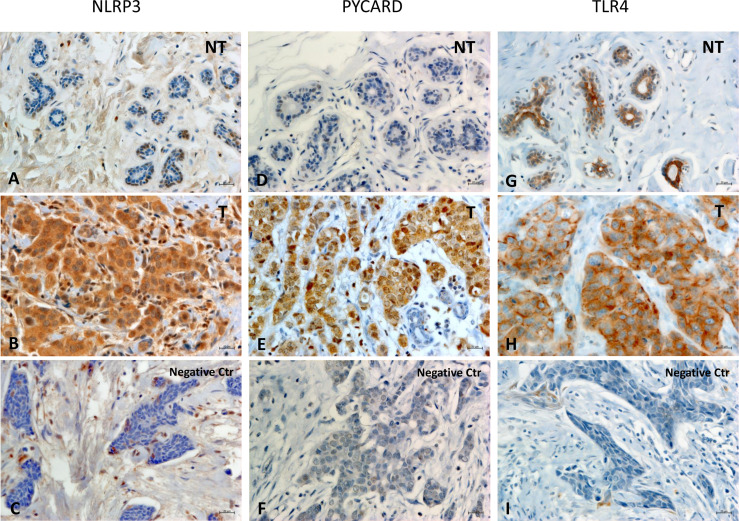
Representative images of immunohistochemical staining in Breast Cancer tissues. The panel displays the representative expression of molecular biomarkers in different areas: **(A)** NOD-like receptor protein 3 (NLRP3) expression in Non Tumoral (NT) area; **(B)** NLRP3 expression in Tumoral (T) area; **(C)** negative control for NLRP3 expression; **(D)** Apoptosis-Associated Speck-Like Protein Containing a Pyrin and CARD domain (PYCARD) expression in NT area; **(E)** PYCARD expression in T area; **(F)** negative control for PYCARD expression; **(G)** Toll like receptor 4 (TLR4) expression in NT area; **(H)** TLR4 expression in T area; **(I)** negative control for TLR4 expression; (original magnification, ×400). Scale bar = 20 µm.

**Figures 1A–I** shows examples of the staining pattern of the proteins analyzed by immunohistochemistry.

**Figure 2:**
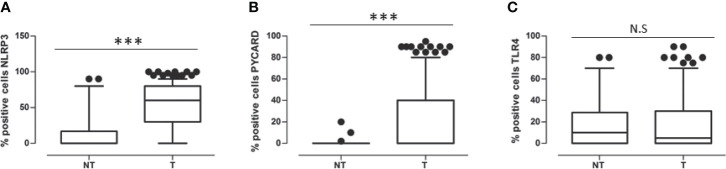
**(A)** Expression levels of NOD-like receptor protein 3 (NLRP3) in Non Tumoral (NT) respect to Tumoral area; **(B)** Expression levels of Apoptosis-Associated Speck-Like Protein Containing a Pyrin and CARD domain (PYCARD) in Non Tumoral (NT) respect to Tumoral area; **(C)** Expression levels of Toll like receptor 4 (TLR4) in Non Tumoral (NT) respect to Tumoral area. Values are expressed as the median (horizontal bold line in each box), with the 5th and 95th percentiles and the furthest points that are not outliers (top and bottom of each ⊺ ⊥ bar). Dot indicates outliers. ***p < 0.001; NS, not significant.

In all the cases, comparison of NLRP3 expression in NT *vs* T tissues showed a statistically significant greater expression in the T areas (p<0.0001). PYCARD expression was also statistically higher in the T than in the NT areas (p<0.0001). By contrast, TLP4 expression was higher in the NT than in the T counterparts, but this difference was not statistically significant (p=0.173) (**Figures 2A–C**).

### Relationship Between Tumor Markers Expression and Clinicopathological Characteristics

**Table 2** shows the relationship between NLRP3, PYCARD and TLR4 and the clinicopathological characteristics.

**Table 2:** Relationship between tumor markers and clinicopathological features.

	NLRP3			PYCARD			TLR4		
	Negative	Positive		Negative	Positive		Negative	Positive	
	N (%)	N (%)	p	N (%)	N (%)	p	N (%)	N (%)	p
**Age**									
≤53	119 (52.4)	53 (50.0)		108 (51.7)	66 (51.2)		101 (48.6)	67 (58.3)	
>53	108 (47.6)	53 (50.0)	0.680	99 (48.3)	63 (48.8)	0.923	107 (51.4)	48 (41.7)	0.095
**Histotype**									
IDC	197 (87.6)	103 (97.2)		178 (87.7)	121 (93.8)		187 (90.8)	103 (89.6)	
ILC	17 (7.5)	2 (1.9)		13 (6.4)	6 (4.7)		12 (5.8)	5 (4.3)	
Other	11 (4.9)	1 (0.9)	**0.020**	12 (5.9)	2 (1.5)	0.116	7 (3.4)	7 (6.1)	0.463
**Tumor size (cm)**									
≤2.0	126 (56.5)	41 (38.7)		104 (51.0)	64 (50.4)		109 (52.9)	51 (44.7)	
>2.0	97 (43.5)	65 (61.3)	**0.002**	100 (49.0)	63 (49.6)	0.917	97 (47.1)	63 (55.3)	0.161
**Node**									
Negative	140 (62.2)	57 (53.8)		131 (64.9)	64 (49.6)		128 (62.1)	59 (51.8)	
Positive	85 (37.8)	49 (46.2)	0.144	71 (35.1)	65 (50.4)	**0.006**	78 (37.9)	55 (48.2)	0.071
**Grade**									
1-2	105 (46.9)	37 (34.9)		86 (42.6)	56 (43.4)		93 (45.1)	39 (34.2)	
3	119 (53.1)	69 (65.1)	**0.040**	116 (57.4)	73 (56.6)	0.881	113 (54.9)	75 (65.8)	0.057
**ER (%)**									
≤10	100 (44.2)	38 (35.1)		95 (46.6)	40 (31.0)		78 (37.7)	59 (51.3)	
>10	126 (55.8)	68 (64.2)	0.148	109 (53.4)	89 (69.0)	**0.005**	129 (62.3)	56 (48.7)	**0.018**
**PgR (%)**									
≤10	127 (56.2)	46 (43.4)		114 (55.9)	59 (45.7)		103 (49.8)	68 (59.1)	
>10	99 (43.8)	60 (56.6)	**0.029**	90 (44.1)	70 (54.3)	0.071	104 (50.2)	47 (40.9)	0.106
**Ki67 (%)**									
≤20	94 (41.8)	37 (35.6)		68 (33.5)	66 (52.0)		90 (44.1)	36 (31.3)	
>20	131 (58.2)	67 (64.4)	0.285	135 (66.5)	61 (48.0)	**0.0009**	114 (55.9)	79 (68.7)	**0.025**
**HER2**									
Negative	197 (87.6)	75 (72.8)		169 (83.7)	102 (80.3)		175 (85.8)	88 (77.2)	
Positive	28 (12.4)	28 (27.2)	**0.001**	33 (16.3)	25 (19.7)	0.438	29 (14.2)	26 (22.8)	0.052
**TNBC**									
No	143 (63.6)	84 (81.5)		131 (64.9)	99 (78.0)		148 (72.6)	70 (61.4)	
Yes	82 (36.4)	19 (18.5)	**0.001**	71 (35.1)	28 (22.0)	0.012	56 (27.4)	44 (38.6)	0.040

p-value of Chi-squared test for the independence of categorical variables. Bold values indicate significance. IDC, Invasive ductal carcinoma; ILC, Invasive lobular carcinoma; ER, Estrogen receptor; PR, Progesterone receptor; HER2/neu, Human epidermal growth factor receptor 2; NLRP3, NOD-like receptor protein 3; PYCARD, Apoptosis-Associated Speck-Like Protein Containing a Pyrin and CARD domain; TLR4, Toll-like receptor 4; TNBC, triple negative breast cancer.

NLRP3 over-expression was observed in invasive ductal carcinoma (IDC; p = 0.020). Higher expression was related to tumor size >2 cm, a higher histological grade (G3) (p = 0.040), PR-positivity (p = 0.029) and human epidermal growth factor receptor 2 (HER2)/neu-positivity (p = 0.001). Higher PYCARD expression showed a significant association with positive node status (p = 0.006), ER-positivity (p = 0.005) and high proliferative activity (Ki67 index) (p = 0.0009). TLR4 was overexpressed in tumors with a high proliferative activity (Ki67 index) (p = 0025) and that were ER-negative (p < 0.018). These TLR4 positive tumors were also associated with a higher histological grade (G3) (p = 0.057) and with positive (HER2)/neu status (p = 0.052). The expression of NLRP3, PYCARD was higher in the non-TNBC phenotype (p=0.001 and p=0.012, respectively). While TLR4 resulted more expressed in the TNBC sub-group (p=0.040).

### Association Between Protein Expressions Analyzed

The Spearman correlation test on continuous variables revealed a direct relation between TLR4 and NLRP3 (r: 0.128; *p* =0.024) and PYCARD expression (r: 0.157; *p* = 0.005) (**Table 3**). Analyzing the dichotomized variables using the χ2 test, a significant frequency of association between TLR4 and NLRP3 expression was found (p= 0.037), while there was no significant association between TLR4 and PYCARD (data not shown).

**Table 3:** Spearman for rank-based correlations between protein expression in breast cancer patients on continuous variables.

	NLRP3		PYCARD	
	r	p-value	r	p-value
**TLR4**	0.128	**0.024**	0.157	**0.005**

Spearman correlation coefficient r (Rho) and p-Value. Bold values indicate significance. NLRP3, NOD-like receptor protein 3; PYCARD, Apoptosis-Associated Speck-Like Protein Containing a Pyrin and CARD domain; TLR4, Toll-like receptor 4.

### Expression of Proteins and Patient Clinical Outcome

Univariate analyses were carried out and the related Kaplan-Meier curves considered for the expression of NLRP3, PYCARD and TLR4 and all clinicopathological characteristics, as dichotomized variables.

**Figure 3:**
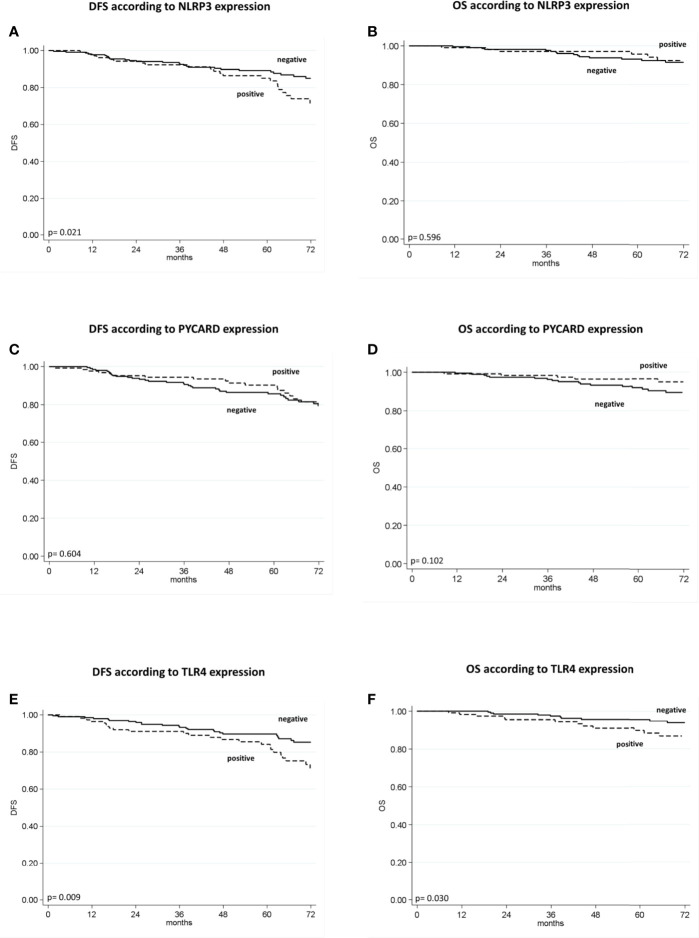
Kaplan–Maier curve analysis and log-rank test. **(A)** Kaplan–Maier curve for disease-free survival (DFS) according to NLRP3 positive versus negative patients (85% *vs.* 89%; 95% CI, 78-92 *vs* 85-93; p = 0.021); **(B)** Kaplan–Maier curve for overall survival (OS) according to NLRP3 positive versus negative patients (96% *vs.* 93%; 95% CI, 92-100 *vs* 90-97; p = 0.596); **(C)** Kaplan–Maier curve for DFS according to PYCARD positive versus negative patients (90% *vs.* 86%; 95% CI, 81-91 *vs* 85-96; p=0.604; **(D)** Kaplan–Maier curve for OS according to PYCARD positive versus negative patients (96% *vs.* 92%; 95% CI, 93-100 *vs* 88-96; p = 0.102); **(E)** Kaplan–Maier curve for DFS according to TLR4 positive versus negative patients (84% *vs.* 90%; 95% CI, 77-91 *vs* 85-94; p = 0.009); **(F)** Kaplan–Maier curve for OS according to TLR4 positive versus negative patients (90% *vs.* 96%; 95% CI, 84-96 *vs* 93-98; p = 0.036).

The patients with high NLRP3 expression had a worse disease-free survival (DFS) than did patients with low NLRP3 expression (85% *vs.* 89%; 95% CI, 78-92 *vs* 85-93; p =0.021). Patients with high TLR4 expression had a worse DFS than did patients with low TLR4 expression (84% *vs.* 90%; 95% CI, 77-91 *vs* 85-94; p = 0.009). No significant differences were observed between patients with high or low PYCARD expression. We also found a significant association between TLR4 expression and overall survival (OS) in that patients exhibiting low TLR4 expression had a better OS than patients with high TLR4 expression (96% *vs* 90%; 95% CI, 93-98 *vs* 84-96; p=0.030) (**Table 4** and **Figure 3**).

**Table 4:** Univariate analysis of DFS (disease-free survival) and OS (overall survival).

Characteristic	N. pts	DFS					OS				
		N. events	5-yrs % DFS (95% CI)	p	HR (95% CI)	p	N. events	5-yrs % OS (95% CI)	p	HR (95% CI)	p
**Overall**	352	60	87 (83-91)	–	–	–	24	94 (91-96)	–	–	–
**Age (years)**											
≤53	182	37	84 (78-90)		1.00		13	92 (88-97)		1.00	
>53	170	23	90 (85-95)	0.159	0.69 (0.41-1.16)	0.161	11	95 (92-99)	0.828	0.91 (0.41-2.04)	0.828
**Histotype**											
IDC	315	53	87 (84-90)		1.00	0.787	21	94 (91-97)		1.00	0.822
ILC	21	5	80 (60-100)		1.38 (0.54-3.50)		2	85 (66-100)		1.58 (0.37-6.76)	
Other	14	2	92 (78-100)	0.787	0.90 (0.22-3.71)		1	92 (78-100)	0.819	1.14 (0.15-8.50)	
**Tumor size (cm)**											
≤2.0	178	18	93 (88-97)		1.00		6	96 (93-99)		1.00	
>2.0	169	42	81 (75-87)	**0.001**	2.45 (1.41-4.26)	**0.001**	18	91 (86-96)	**0.011**	3.11 (1.23-7.85)	**0.016**
**Node**											
Negative	208	28	89 (85-93)		1.00		12	94 (90-97)		1.00	
Positive	141	31	84 (77-90)	**0.025**	1.78 (1.07-2.97)	**0.027**	12	93 (88-98)	0.284	1.54 (0.69-3.44)	0.288
**Grade**											
1-2	150	10	96 (93-99)		1.00		3	98 (95-100)		1.00	
3	199	49	80 (74-86)	**<0.0001**	3.65 (1.84-7.24)	**0.0002**	21	90 (86-95)	**0.002**	5.29 (1.58-17.76)	**0.007**
**ER (%)**											
≤10	147	37	80 (74-87)		1.00		15	90 (86-95)		1.00	
>10	204	22	92 (88-96)	**0.006**	0.48 (0.28-0.82)	**0.007**	9	96 (93-99)	0.079	0.48 (0.21-1.11)	**0.085**
**PgR (%)**											
≤10	185	42	83 (77-88)		1.00		18	91 (87-95)		1.00	
>10	166	17	92 (87-97)	**0.020**	0.51 (0.29-0.91)	**0.023**	6	96 (93-100)	**0.052**	0.41 (0.16-1.04)	**0.060**
**Ki67 (%)**											
≤20	141	13	95 (91-99)		1.00		1	99 (97-100)		1.00	
>20	207	44	83 (77-88)	**0.013**	2.16 (1.16-4.02)	**0.015**	23	90 (86-95)	**0.0004**	14.79 (2.00-109.58)	**0.008**
**HER2**											
Negative	289	46	87 (83-91)		1.00		18	94 (91-97)		1.00	
Positive	58	10	87 (78-96)	0.471	1.29 (0.65-2.55)	0.472	6	92 (85-100)	0.171	1.89 (0.75-4.76)	0.179
**TNBC**											
No	237	29	91 (87-95)		1.00		13	95 (92-98)		1.00	
Yes	110	27	80 (72-87)	0.037	1.75 (1.03-2.99)	0.040	11	90 (84-96)	0.232	1.63 (0.73-3.65)	0.236
**NLRP3**											
Negative	227	31	89 (85-93)		1.00		16	93 (90-97)		1.00	
Positive	106	24	85 (78-92)	**0.021**	1.88 (1.09-3.24)	**0.023**	6	96 (92-100)	0.596	0.78 (0.30-1.98)	0.597
**PYCARD**											
Negative	205	37	86 (81-91)		1.00		18	92 (88-96)		1.00	
Positive	129	19	90 (85-96)	0.604	0.86 (0.50-1.50)	0.604	5	96 (93-100)	0.102	0.45 (0.17-1.20)	0.112
**TLR4**											
Negative	208	26	90 (85-94)		1.00		10	96 (93-98)		1.00	
Positive	115	28	84 (77-91)	**0.009**	2.01 (1.18-3.43)	**0.010**	13	90 (84-96)	**0.030**	2.41 (1.06-5.51)	**0.036**

Median follow-up: 67 months (range 1-199).

Bold values indicate significance. HR, Hazard-ratio; IDC, Invasive ductal carcinoma; ILC, Invasive lobular carcinoma; ER, Estrogen receptor; PR, Progesterone receptor; HER2/neu, Human epidermal growth factor receptor 2; NLRP3, NOD-like receptor protein 3; PYCARD, Apoptosis-Associated Speck-Like Protein Containing a Pyrin and CARD domain; TLR4, Toll-like receptor 4; TNBC, triple negative breast cancer.

Univariate analysis indicated a worse DFS rate for tumor size >2 cm than for tumor size ≤2 cm (81% *vs.* 93%; 95% CI, 75-87 *vs* 88-97; p = 0.001), positive compared to negative nodal status (84% *vs.* 89%; 95% CI, 77-90 *vs* 85-93; p = 0.025), high (G3) compared to low (G1–2) histological grade (80% *vs.* 96%; 95% CI, 74-86 *vs* 93-99; p < 0.0001), high compared to low Ki67 expression (83% *vs.* 95%; 95% CI, 77-88 *vs* 91-99; p =0.013). Conversely, ER and PR positivity showed a better DFS than did ER and PR negativity (92% *vs.* 80%; 95% CI, 88-96 *vs* 74-87; p = 0.006; 92% *vs.* 83%; 95% CI, 87-97 *vs* 77-88; p = 0.020 respectively). The TNBC patients had a worse disease-free survival (DFS) than no-TNBC patients (80% vs. 91%, 95% CI 72-87 *vs* 87-95, p=0.037). Poor OS was observed for tumor size >2 cm compared to tumor size ≤2 cm (91% *vs.* 96%; 95% CI, 86-96 *vs* 93-99; p = 0.011), high (G3) compared to low (G1-2) histological grade (90% *vs.* 98%; 95% CI, 86-95 *vs* 95-100; p = 0.002), and high compared to low Ki67 expression (90% *vs.* 99; 95% CI, 86-95 *vs* 97-100; p = 0.0004). A statistical trend was found in patients with positive PR expression who had a better OS (96 *vs* 91; 95% CI, 93-100 *vs* 87-95; p=0.052), (**Table 4**).

According to the Cox proportional hazard regression model, multivariate analysis showed that TLR4 is independent prognostic factors, with high expression associated to a shorter DFS (Hazard Ratio (HR) = 2.03, 95% Confidence Interval (CI) 1.16–3.57, p = 0.014) and a shorter OS (HR = 2.54, 95% CI 1.06–6.05, p = 0.036). High NLRP3 expression showed a slight, albeit not significant, association with DFS (HR = 1.75, 95% CI 0.98–3.15, p = 0.06) (**Table 5**).

**Table 5:** Multivariate analysis of DFS (disease-free survival) and OS (overall survival).

	DFS		OS	
	HR (95% CI)	p	HR (95% CI)	p
**% PYCARD**				
Negative (<20 cutoff)	1.00		1.00	
Positive (≥20)	0.78 (0.43-1.41)	0.414	0.51 (0.18-1.40)	0.190
**% TLR4**				
Negative (<20 cutoff)	1.00		1.00	
Positive (≥20)	2.03 (1.16-3.57)	**0.014**	2.54 (1.06-6.05)	**0.036**
**% NLRP3**				
Negative (<80 cutoff)	1.00		1.00	
Positive (≥80)	1.75 (0.98-3.15)	**0.060**	0.79 (0.30-2.09)	0.640

Bold values indicate significance. HR, Hazard-ratio; NLRP3, NOD-like receptor protein 3; PYCARD, Apoptosis-Associated Speck-Like Protein Containing a Pyrin and CARD domain; TLR4, Toll-like receptor 4.

The same investigation has been carried out in the TNBC subgroup (protein expression profiling, relationship with clinicopathological characteristics, protein association and patient clinical outcome), but not substantial relations have been found in this sub-set (Data not shown).

## Discussion

Despite the great interest it has garnered over the last few years, the molecular mechanism of NLRP3 inflammasome action remains poorly understood, especially its role in cancer. Aberrant activation of the NLRP3 inflammasome has been observed in several malignancies (13). It has recently been demonstrated that up-regulation of NLRP3 inflammasome expression in human breast Cancer-Associated Fibroblasts (CAFs) is a steppingstone to cancer progression and metastasis (25). NLRP3 activation in both cancer cells and stromal components could result in a cumulative mechanism creating a tumor microenvironment favorable to cancer progression. The identification of new biomarkers linked to inflammasome action could help in the prognosis of BC and the development of new targeted BC therapies to support traditional treatments.

In this study we focused on NLRP3 inflammasome activation in the tumor counterparts to examine the activity of this complex in BC, evaluate its possible contribution to prognosis and provide indications for future combination therapies.

In our BC cohort, NLRP3 and PYCARD expression was higher in the tumor samples than in the non-cancerous counterparts, thus confirming inflammasome involvement in establishing a tumor-associated microenvironment to support cancer progression (26). More than 50% of our patients presented high tumor grade (G3) and high proliferative activity (Ki67), underlining the aggressiveness of these tumors. Clinical analysis revealed that NLRP3 and PYCARD expression was strongly associated with the presence of several more aggressive clinicopathological factors, such as tumor size, histological grade and Ki67 index and pointed to the contribution of both proteins to BC progression *via* their relationship with the expression of receptors and factors closely associated with tumor growth. The expression of NLRP3, PYCARD was higher in the non-TNBC phenotype. In the last years different authors have described the negative regulation of ER and PgR on NLRP3 inflammasome activation, demonstrating a hormonal modulation of inflammasome platform in different diseases (27–30).

Recent studies reported higher NLRP3 and PYCARD protein expression in cancer tissues than in adjacent normal tissues from patients with laryngeal squamous cell carcinoma (LSCC) (31) and colorectal cancer (CRC) (32). The authors also correlated NLRP3 inflammasome expression to the patients’ clinicopathological characteristics (31). The NLRP3 inflammasome appears to be involved in tumor aggressiveness, given its overexpression in the tumor areas and its association with greater tumor size, higher histological grade and positive node and receptor status. NLRP3 inflammasome activation is related to nuclear factor-kB (NF-kB) activation by TLR signaling and is a key link between inflammation and cancer (31, 33–35). We examined TLR4 expression and its interaction with NLRP3 in our BC cohort. TLR4 was associated both to tumors with high proliferative activity and TNBC phenotype, as already reported in experimental evidence showing its involvement in BC progression, invasion and drug resistance by initiating and supporting an inflammatory environment (36–39). TLR4 was directly related with NLRP3 and PYCARD demonstrated a positive synergistic correlation supporting malignant phenotypes, although the correlation factor was not strong. Reciprocal crosstalk between the NLRP3 inflammasome and TLR4 is not a surprise in other malignancies (40, 41) but for the first time, we found a direct indication of their interaction in our BC patients.

In the Kaplan-Meier survival analysis, the sub-group of patients with high NLRP3 expression had a worse 5-year survival rate than did patients with low NLRP3 expression. The same trend was also observed in patients overexpressing the TLR4 protein, as reported by other authors (42, 43).

This finding is very interesting as it indicates that the expression levels of NLRP3 inflammasome members may be a risk factor for BC progression. Inflammasomes have been described as cancer hallmarks and their suppressive activity on the immune system is well-known (14, 44). NLRP3 could also support tumor progression-related phenomena such as EMT (45–47), cancer stem cells renewal activation (48), and an increase in MDCSCs (49).

The multivariate analysis indicated that TLR4 is independent prognostic factors with high expression associated to a shorter DFS and OS in BC. This is not surprising considering that TLR4 is related to cancer aggressiveness and poor clinical outcome (20, 42, 43, 50–52). NLRP3 expression showed a slight, albeit not significant, association with DFS and this association to the clinical effects of BC is a new compelling point. Its contribution to the onset and progression of malignant phenotypes has been reported for oral squamous cell carcinoma (53) and pancreatic cancer (54). A recent study in a CRC model showed that NLRP3-positive patients had a poor prognosis, and that NLRP3 was an independent prognostic factor for the survival of patients (32).

## Conclusions

In conclusion, we found that TLR4 expression is an independent prognostic factors and highlighted for the first time that high expression of NLRP3 is linked to a poor prognosis in BC patients and that it could be a good prognostic factor. The NLRP3 signaling pathway is closely related with the TLR4 and both could have a synergic role in BC progression.

Further, these results suggest that NLRP3 and TLR4 could be new targets in combination therapies to increase and enhance treatment options for BC patients. Prospective trials to validate these findings and further elucidate the clinical utility of these biomarkers will be warranted for BC patients starting new systemic treatments.

## Publisher’s Note

All claims expressed in this article are solely those of the authors and do not necessarily represent those of their affiliated organizations, or those of the publisher, the editors and the reviewers. Any product that may be evaluated in this article, or claim that may be made by its manufacturer, is not guaranteed or endorsed by the publisher.

## Data Availability Statement

The raw data supporting the conclusions of this article will be made available by the authors, without undue reservation.

## Ethics Statement

The study was approved by the Ethics Committee of the Istituto Tumori “Giovanni Paolo II” with document no. 234/CE of 13 November 2017. Written informed consent for participation was not required for this study in accordance with the national legislation and the institutional requirements.

## Author Contributions

Conceptualization: AM. Methodology: MS, AC, FB, and MP. Data acquisition: CS, MS, and AC. Data analysis and interpretation: AM, ES, FG, and CS. Writing original draft: AM and CS. Writing, review, and editing: all authors. Funding acquisition: AM and FZ. Supervision: AM. All authors contributed to the article and approved the submitted version.

## Funding

This research was funded by the Italian Ministry of Health, “Ricerca Corrente 2021,” Del. 153/2021.

## Conflict of Interest

The authors declare that the research was conducted in the absence of any commercial or financial relationships that could be construed as a potential conflict of interest.

## Publisher’s Note

All claims expressed in this article are solely those of the authors and do not necessarily represent those of their affiliated organizations, or those of the publisher, the editors and the reviewers. Any product that may be evaluated in this article, or claim that may be made by its manufacturer, is not guaranteed or endorsed by the publisher.
