# Supplementary material for: Prognostic Value of NLRP3 Inflammasome and TLR4 Expression in Breast Cancer Patients
Source: Front Oncol. 2021 Sep 2;11:705331. doi: 10.3389/fonc.2021.705331 (PMC8443770; doi:10.3389/fonc.2021.705331)
Supplement: Supplementary file 1 [file Table_1.docx]

Supplementary Material

# Supplementary Table 1. Characteristics, diluitions and localization of used antibodies

| **Antibody target** | **Antibody type** | **Antigen retrieval buffer** | **Dilution** | **Supplier** | **Code** | **Localization** |
| --- | --- | --- | --- | --- | --- | --- |
| NLRP3 | Rabbit polyclonal | EDTA | 1:50 | abcam | Ab214185 | cytoplasm |
| Pycard | Mouse monoclonal | Citrate | 1:600 | ThermoFisher | MA5-26363 | cytoplasm |
| TLR4 | Mouse monoclonal | EDTA | 1.100 | abcam | Ab22048 | membrane/cytoplasm |

Citrate buffer at pH 6; ethylenediaminetetraacetic acid (EDTA) at pH 7.8. NLRP3: NOD-like receptor protein 3; PYCARD: Apoptosis-Associated Speck-Like Protein Containing a Pyrin and CARD domain; TLR4: Toll-like receptor 4.

**
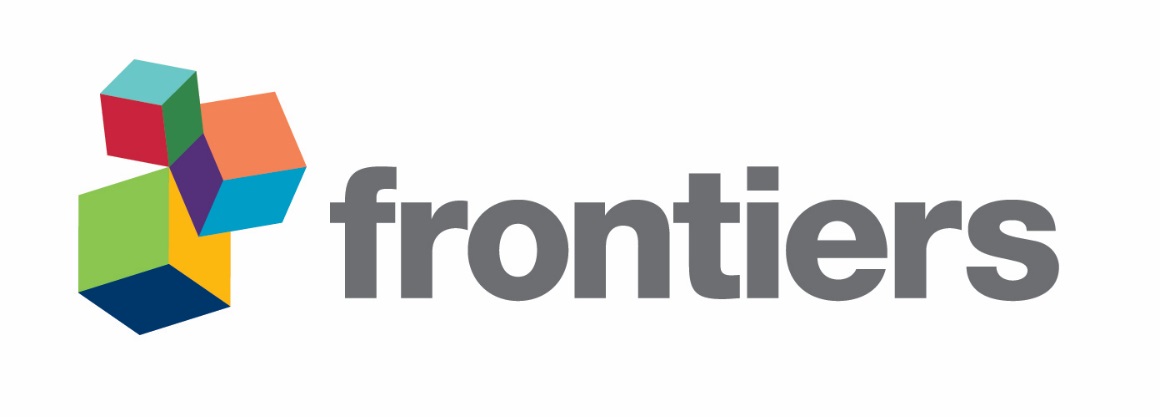
**
